# How to approach and treat viral infections in ICU patients

**DOI:** 10.1186/1471-2334-14-321

**Published:** 2014-11-28

**Authors:** Theodoros Kelesidis, Ioannis Mastoris, Aliki Metsini, Sotirios Tsiodras

**Affiliations:** Department of Medicine, Division of Infectious Diseases, David Geffen School of Medicine at UCLA, Los Angeles, CA USA; 4th Department of Internal Medicine, Attikon University Hospital, National and Kapodistrian University of Athens School of Medicine, 1 Rimini Street, GR-12462 Haidari, Athens, Greece

## Abstract

Patients with severe viral infections are often hospitalized in intensive care units (ICUs) and recent studies underline the frequency of viral detection in ICU patients. Viral infections in the ICU often involve the respiratory or the central nervous system and can cause significant morbidity and mortality especially in immunocompromised patients. The mainstay of therapy of viral infections is supportive care and antiviral therapy when available. Increased understanding of the molecular mechanisms of viral infection has provided great potential for the discovery of new antiviral agents that target viral proteins or host proteins that regulate immunity and are involved in the viral life cycle. These novel treatments need to be further validated in animal and human randomized controlled studies.

## Introduction

The prevalence of viral diseases has increased due to the availability of modern diagnostic tests that allow rapid detection of viruses [1]. Viral diseases may additionally be associated with significant morbidity and mortality as is the case with some emerging viral diseases, such as the Middle East Respiratory Syndrome coronavirus or avian influenza [2, 3]. Patients with severe viral infections are often hospitalized in intensive care units (ICUs); on the other hand recent studies have underlined the frequency of virus detection in ICU patients [4–6]. The majority of viral infections that require ICU care involve the respiratory tract or the central nervous system. However, other organ systems, such as the gastrointestinal tract, may be severely affected by viruses and require support or close monitoring. The reported incidence of viral infections reported in the ICU varies widely across studies and geographic regions and has changed over the recent years based on the epidemiology of emerging viral infections such as human metapneumovirus and adenovirus infections [7, 8]. Improved molecular detections methods have also significantly changed the epidemiology of viral infections in the ICU over the last years [7]. Multi-institutional databases and time-series models may be useful tools to characterize and forecast the burden of severe viral infections at the local and institutional levels [9, 10]. Clinical signs and symptoms are rarely sufficient to make a specific diagnosis of a viral infection. Often a combination of the appropriate clinical syndrome together with epidemiologic clues but more importantly specific laboratory tests is used to reach the diagnosis [11]. Viral infections can cause severe morbidity and mortality in certain hosts such as immunocompromised patients (Table 1) [12–52]. Herein, we review the literature on the role of viruses in ICU in adults [excluding Human Immunodeficiency Virus (HIV)] with a focus on treatment of these infections.

**Table 1:** **Etiologies and treatment of viral syndromes in the ICU**

Syndrome/presentation	Common viruses	Treatment
**RESPIRATORY FAILURE**		
**Hypoxic respiratory failure-pneumonia**	**Hypoxic respiratory failure:** *Influenza A and B, RSV A and B, coronavirus, Severe Acute Respiratory Syndrome (SARS), Middle East Respiratory Syndrome coronavirus, Adenovirus, cytomegalovirus, Varicella, HSV, Parainfluenza 1-4, Metapneumovirus, measles* especially in immunocompromised patients	**Supportive:** adequate oxygen delivery
	**VAP:** HSV, CMV, Mimivirus	
**Hypercapnic-hypoxic respiratory failure**	**Hypercapnic-hypoxic respiratory failure:** *Influenza A and B, coronavirus, rhinovirus, Parainfluenza 1-4, RSV A and B*	**Antivirals:**
Asthma/COPD exacerbation		Neuraminidase inhibitors (NAIs) (Oseltamivir, Zanamivir, peramivir, Laninamivir) [12, 13]. For resistant influenza viruses may consider combination therapy of NAI with ribavirin and/or novel antivirals such as Favipiravir [14, 15]
Adult Respiratory Distress Syndrome (ARDS)	**ARDS:** *Influenza virus, Hantavirus* [Hantavirus pulmonary syndrome (HPS)], *varicella, herpes simplex virus, SARS,* MERS-CoV	Ribavirin for RSV in immunocompromised patients and children [16–18] and may also be considered for other viruses such as in SARS [25] or MERS-CoV - lopinavir in combination regimens has also been used
Without lung disease (restrictive disease): Guillain-Barré syndrome (GBS)	**GBS:** *HSV, VZV, CMV, EBV, Influenza, Hantavirus acute and chronic hepatitis B,* Rare causes: *West Nile virus, Parvovirus B19, Hantavirus, rubella, dengue*	Acyclovir for VZV pneumonitis (limited efficacy it is still widely recommended as early primary therapy) [19]
		Ganciclovir for CMV pneumonitis in solid organ transplant patients appears to reduce morbidity [20]
		**Corticosteroids:** For influenza [21–23], SARS[24, 25] and VZV pneumonitis [26] to reduce inflammatory tissue injury in severe pneumonia
		**Immunotherapies:** Palivizumab is approved for high-risk pediatric patients with RSV infection [18]; IVIG for certain respiratory viruses including influenza [27, 28] and GBS, plasma exchange for GBS. Combinations of ganciclovir with immunoglobulin or cytomegalovirus immunoglobulin may be of value in patients with bone marrow transplants and CMV pneumonitis [29, 30]
		**Others:** Vitamin A for severe measles [31]
**Neurological syndromes**		
Encephalitis, meningitis, meningoencephalitis, myelitis, polyradiculo-neuropathy, Guillain-Barré syndrome (GBS) Reyes syndrome, subacute sclerosing panencephalitis, postinfectious acute disseminated encephalomyelitis (ADEM) [32]	*HSV* (40% to 50% of encephalitis cases where a cause is determined, and 10% to 20% overall [32]*VZV* (the most common cause of encephalitis among immunocompromised patients and the second most common viral cause of sporadic encephalitis not occurring during an outbreak)	**Supportive:** Treatment of neurologic (eg, cerebral edema, high intracranial pressure, and seizures) and systemic (eg, hypoxemia, low cerebral perfusion pressure, and fever) complications
**Clinical presentation:** usually as altered mental status, seizures, coma, neuropathies	*Enteroviruses (Enterovirus 71, Coxsackie, Echovirus, poliovirus*: as a group, enteroviruses) are collectively the third most common cause of sporadic viral encephalitis and the most common cause of aseptic meningitis	**Antivirals:**Acyclovir: Early aggressive antiviral therapy with acyclovir for HSV, VZV improves mortality and reduces subsequent cognitive impairment
	*Arboviruses (JEV, WNV, TBEV, MVEV, LCEV, SLEV, EEEV:* the most common pathogens to cause encephalitis that is restricted to certain geographic regions)	
	*Influenza* (encephalitis is very uncommon complication of seasonal influenza infections but because influenza itself is common 4-19% of patients with severe or fatal H1N1 reported neurologic complications	Ganciclovir: CMV encephalitis
	Other viruses: *West Nile virus, CMV, mumps, measles, rubella, rabies, JC virus (PML), acute HIV infection)*	Foscarnet: HHV-6, combination therapy with foscarnet and ganciclovir is recommended for CMV encephalitis
		Oseltamivir: Severe influenza
		Pleconaril: severe Enterovirus infections
		**Corticosteroids:** Complicated HSV encephalitis (data based on retrospective studies), VZV encephalitis (for inflammatory vasculopathy), uncomplicated zoster (variable results), severe influenza, WNV (case report) [33], postinfectious encephalitis
		**Immunotherapies:** Immunomodulatory therapy with either intravenous immune globulin or plasma exchange for patients with postinfectious encephalitis who fail corticosteroid treatment (data based on case series) or for WNV encephalitis (Case reports) [34, 35].
		**Others:** Vitamin A for severe measles [31]
**Virus related shock**		
**Cardiogenic shock**	*Enteroviruses (Enterovirus 71, Coxsackie viruses group A and B, Echovirus), Influenza, Adenovirus, Parvovirus, RSV, CMV, HIV-1, hepatitis A and C viruses, vaccinia virus (after smallpox vaccine)*	**Supportive**
Myocarditis		**Antivirals:**
		Rifampin: For RSV myocarditis [36]
		Pleconaril: severe Enterovirus infections
		Oseltamivir: Severe influenza
		ART: HIV-1
		**Corticosteroids:** do not reduce mortality (data based on small RCT of poor quality) [37]
		**Immunotherapies:** IVIG (data based on in vitro data, case series, limited RCT) [38–40]. Combination therapy of IVIG with rifampin has been described in case series [36]
		**Others:** Herbal medicines [41], mechanical ventricular assist devices until resolution or cardiac transplantation is available, novel therapies e.g pleconaril
**Distributive shock-Hemorrhagic fever**	*Arenaviruses (South American HF-Junin; Lassa Fever), Bunyaviruses (Rift valley fever, Chrimean Congo HF-CCHF), HF with renal syndrome, Hantavirus, Filoviruses (Ebola, Marburg), Flaviviruses (Yellow fever, Dengue HF)*	**Supportive:** adequate oxygen delivery, blood products.
**Clinical presentation:** Febrile illnesses, headache, myalgia, nausea, vomiting and diarrhea are frequent. Hemorrhagic features, disseminated intravascular coagulopathy (DIC), multiple organ system failure and death ensue.		**Passive transfer of antibodies** (plasma, IVIG) may be of value in Bunyaviruses [45], Junin virus [42], Lassa virus [43], Hantavirus HF [17, 44], Flaviviruses (Yellow fever, Dengue HF) [45–47]
		**Antivirals:** ribavirin for CCHF [17, 48], Lassa virus [17, 49], Hantavirus HF [17, 44]
		Ribavirin plus interferon may be considered for Lassa virus [50]
**Hypovolemic/distributive shock in the setting of acute liver failure secondary to viral hepatitis**	*Hepatitis A, B, C, D, E, G, herpes group (CMV, HSV and Epstein Barr virus), adenovirus and influenza virus*	**Supportive:** hemodynamic management, ventilation, prevention and treatment of hemorrhage, dialysis, therapy of co-existent sepsis and electrolyte disturbance, and management of intracranial pressure
**Clinical presentation:** Nausea and vomiting with progression to encephalopathy and coma; may be new onset or acute decompensation of chronic liver failure due to viral hepatitis/cirrhosis		**Orthotopic liver transplantation**
		**Antivirals** (may be used for acute flare up of chronic viral hepatitis e.g. in immunocompromised patients.
**Hypovolemic/distributive shock in the setting of acute pancreatitis**	*Mumps* (the most common virus associated with pancreatitis, occurring even in the absence of parotitis), *Enteroviruses (Coxsackie B), cytomegalovirus, varicella zoster, HSV-1, Epstein-Barr virus, influenza A, Parainfluenza, adenovirus, measles.* In fulminant hepatic failure due to hepatitis A (HAV) or hepatitis E (HEV) pancreatitis occurs in up to 34% of the cases [51]	**Supportive**
		**Antivirals**
		Oseltamivir: Severe influenza
		Pleconaril: severe Enterovirus infections
		Acyclovir: VZV
**Shock in the setting of adrenal insufficiency caused by viral infection (rare)**	CMV in HIV-1 infection [52]	Treatment of CMV itself is generally not warranted, unless there is evidence of CMV disease elsewhere. However, it is critical to treat the underlying human immunodeficiency virus infection with antiretroviral agents to attempt immune restitution [52]
**Rhabdomyolysis**	Influenza A and B, Parainfluenza virus, CMV, EBV, VZV, measles, adenovirus, enteroviruses	**Supportive**
		**Antivirals**
		Oseltamivir: Severe influenza
		Pleconaril: Severe Enterovirus infections
		Acyclovir: VZV
		Ganciclovir: CMV
**Special Immunocompromised host**		
Trauma/Burn	HSV, CMV	Supportive, antivirals, corticosteroids
Pregnancy	HSV, VZV, CMV, Influenza virus	Supportive, antivirals
Transplantation	CMV, EBV [post-transplant lymphoproliferative disorder (PTLD)], VZV, HSV, HHV-6 and HHV-8, RSV, Influenza A and B, BK virus, Adenovirus	Supportive, antivirals, immunotherapies (for example donor lymphocyte infusions and anti-CD20 antibody for PTLD), experimental therapies

*Abbreviations*: *ADEM* acute disseminated encephalomyelitis, Adult Respiratory Distress Syndrome (ARDS), *CMV* Cytomegalovirus, *CCHF* Chrimean Congo Hemorrhagic Fever, *COPD* Chronic Obstructive Pulmonary Disease, *DIC* disseminated intravascular coagulopathy, *EBV* Epstein Barr virus, Guillain-Barré syndrome (GBS), *HAV* hepatitis A virus, *HBV* hepatitis B virus, *HCV* hepatitis C virus, or *HEV* hepatitis E virus, *HIV* human immunodeficiency virus, *HHV-6* Herpes Virus 6, *HHV-8* Herpes Virus 8, *HF* Hemorrhagic Fever, *HSV* Herpes Simplex Virus, *NAIs* Neuraminidase inhibitors, *ICU* Intensive Care Unit, *JEV* Japanese Encephalitis Virus, *MVEV* Murray Valley encephalitis virus, *PTLD* post-transplant lymphoproliferative disorder, *RCT* Randomized Controlled trials, *RSV* Respiratory Syncytial Virus, *SARS* Severe Acute Respiratory Syndrome, *TBEV* tick-borne encephalitis virus, *SLEV* St. Louis Encephalitis Virus, *VZV* Varicella-Zoster Virus, *WNV* West Nile virus.

## Review

### Respiratory infections

In recent years, viruses have been identified as an increasingly frequent cause of community-acquired pneumonia (CAP) [53], because of the availability of new diagnostic tools, such as Polymerase Chain Reaction (PCR). On the other hand the emergence of the pandemic influenza virus in 2009 as well as the emergence of viruses with pandemic potential such as the avian influenza viruses or new coronaviruses has emphasized the role of viruses in severe community acquired pneumonia in places where these viruses are endemic [54]. Viral nosocomial pneumonia [hospital-acquired, healthcare-associated pneumonia (HCAP) or ventilator-associated pneumonia (VAP)] have been described but the pathogenicity and the roles of viruses recovered from the lower respiratory tract in patients with pneumonia remains controversial. Severe viral infections such as influenza, severe acute respiratory syndrome (SARS) may cause respiratory failure which may rapidly progress to acute respiratory distress syndrome (ARDS) and multi-organ failure [55–58]. Except for pneumonia, acute respiratory failure can occur in patients with chronic obstructive pulmonary disease (COPD) and lead to hospitalization and the need for mechanical ventilation [55–58]. In addition, viruses can cause ARDS and neurogenic respiratory failure (for example through development of Guillain-Barré Syndrome) [55–58].

### Causes of viral pneumonia

#### Respiratory viruses are the most common cause of viral CAP

Although severe community-acquired pneumonia is usually caused by bacteria, viruses account for approximately 3-10% of cases in large series [59–65]. The most common cause of viral pneumonia in adults is influenza virus type A and B [32, 53, 65–73]. Immunocompromised patients are more likely to have viral pneumonias caused by respiratory syncytial virus (RSV), cytomegalovirus (CMV), herpes simplex virus (HSV), varicella-zoster virus (VZV), adenovirus and rarely measles (21-35). Recent molecular diagnostic methods have significantly changed the epidemiology of viral pneumonias in the ICU over the last years with the increasing detection of viruses such as human metapneumovirus and adenovirus infections [7, 8]. Radiographic findings are variable and not virus specific; an “atypical” pneumonia presentation is often seen in otherwise healthy individuals while on the other hand severe lobar or bilateral pneumonia can be seen in immunocompromised hosts. All the reported respiratory viruses can cause severe pneumonia with acute respiratory distress syndrome (ARDS) requiring mechanical ventilation, but the frequency of this complication is not known [55–58].

#### Respiratory viruses may be the cause of HCAP

Viral pneumonias may be nosocomially acquired, especially during peak respiratory periods and in immunocompromised patients [74–76]. In a recent retrospective study, 34% of the 134 HCAP patients had at least one respiratory virus recovered either in the lower respiratory tract or the nasopharyngeal swab [77], with the most frequent being rhinovirus, parainfluenza virus, human Metapneumovirus and influenza. Patients with viral HCAP or bacterial VAP had the same mortality rate [77].

#### Mostly latent viruses, particularly Herpesviridae, are identified in patients with VAP

Although data on viral nosocomial pneumonia are scarce, the role of respiratory viruses as a cause of nosocomial pneumonia is probably limited. In two studies in ICU patients, <5.5% of mechanically ventilated patients with VAP had a respiratory sample positive for respiratory viruses [6, 72, 78] and in many of these cases the mechanical ventilation duration before virus detection may have indicated carriage before ICU admission. Latent viruses such as Herpesviridae including herpes simplex virus (HSV) and cytomegalovirus (CMV) are known to be a cause of pneumonia or systemic disease in immunocompromised patients [79] but are often reactivated in non-immunocompromised ICU patients. ICU patients are known to experience immunoparalysis since an initial proinflammatory is followed by an anti-inflammatory response; this immunological state is responsible for nosocomial infections and latent virus reactivation [80, 81]. In most patients, viral detection reflects viral reactivation without lung parenchymal involvement. However, viral lung disease may develop, usually in patients with prolonged mechanical ventilation [6, 82, 83]. Mimivirus, an emergent virus, has also been described as a possible cause for nosocomial VAP [84–88]. Although patients with high HSV and CMV viremia often have worse prognosis, the exact significance of detection of HSV, CMV or mimivirus in the lower respiratory tract of ventilated non-immunocompromised ICU patients is unclear [4–6, 72, 82, 83, 89]. Further clinical research work is needed to elucidate the role of these viruses in the pathogenesis of nosocomial viral pneumonia.

### Treatment of viral respiratory infections

#### Treatment of viral CAP remains largely supportive

Influenza is the only virus for which Food and Drug Administration (FDA)-approved therapeutic agents are available for adults. The most effective measure against influenza remains vaccination, particularly for the elderly or high-risk individuals [90]. Antivirals for the treatment of influenza include the M2 channel inhibitors and the neuraminidase inhibitors [91]. Although treatment with neuraminidase inhibitors (oseltamivir or zanamivir) is recommended in all patients with suspected or confirmed influenza requiring hospitalization [92] their use in non-severe influenza could be more harmful than beneficial because of the possibility of selection of resistant mutants [93]. Thus, it would be appropriate to use them only for patients with severe disease presentation, for example, severe pneumonia, requiring mechanical ventilation or patients at high risk for influenza associated complications e.g immunocompromised individuals. Alternatively it can be used in all suspect cases in areas endemic for a strain with high mortality e.g. an avian influenza strain. Higher dosing regimens such as 150 mg twice daily may be safe and well tolerated [94–98], have been used to treat seriously ill patients [58, 99, 100] and may have a benefit for treatment of Influenza B [101], some influenza A strains with reduced susceptibility [12, 102–106] as well as infection sites with limited drug penetration (eg, central nervous system, as in some H5N1 cases) [96, 98, 107, 108]. However, overall supportive evidence is lacking [91, 94, 95, 99, 101, 102, 109–111] and antiviral resistance may emerge even with higher doses of oseltamivir [112].

#### Novel antivirals can be considered for treatment of respiratory viral infections

Two new neuraminidase inhibitors have recently been described: peramivir and laninamivir octanoate. Peramivir, which can be given as a single intravenous dose, was authorized for a short period by the US Food and Drug Administration (FDA) for emergent intravenous use in hospitalized patients with the 2009 H1N1 pandemic influenza virus [113]. Laninamivir is given as a single inhaled dose for the treatment of seasonal influenza in adults and may also treat oseltamivir-resistant virus [113]. In addition, new therapeutics for the treatment of influenza A virus infections are under development [13–15, 18, 28, 39, 50, 114–195]. In this regard, the drug, favipiravir (T-705) has been shown to inhibit a variety of influenza viruses, including highly pathogenic avian influenza H5N1 viruses. Finally, numerous antivirals such as entry inhibitors, nucleoside analogues such as cidofovir, viral enzyme inhibitors (such as terminase and helicase enzyme inhibitors), and translation inhibitors may be utilized in an off-label indication for treatment of viral infections [13, 113].

#### Combination antiviral therapy can be used for treatment of resistant influenza

Except for HIV, hepatitis C and hepatitis B, combination drug therapies are not established for other viruses, such as HSV and influenza. Triple and dual drug combinations may be synergistic in their antiviral action [196]. The efficacy of oseltamivir-zanamivir combinations for seasonal influenza was established in a randomized controlled clinical study [188]. However, clinical antagonism between oseltamivir and zanamivir was suggested in another study [188, 193].

#### Other therapies for treatment of influenza

Low-dose systemic corticosteroids may be used for septic shock related to severe influenza [58] since evidence from RCTs suggests that corticosteroids may be associated with delayed clearance of viruses [21–23] and invasive fungal infections [197]. Case control studies and a RCT suggested that plasma and hyperimmune globulin have demonstrated favorable responses in patients with severe avian influenza A (H5N1) and H1N1pdm09 infection compared with controls [27, 28, 198]. Further evaluation of novel treatments with RCTs is needed.

### Viral infections of the nervous system in the ICU

Several viruses may infect the central nervous system (CNS) and cause inflammation of the meninges and brain parenchyma causing meningitis, encephalitis, seizures, coma and respiratory failure, secondary to aspiration, neuromuscular weakness and increasing atelectasis [32].

#### The etiology of meningoencephalitis is often not identified

Several viruses may cause infectious and postinfectious complications in the nervous system (Table 1). Despite advances in molecular techniques a specific cause is found in less than half of the cases [32].

#### Modern ICU care has significantly improved prognosis of viral nervous system infections

Acyclovir has significantly improved the prognosis of HSV encephalitis. Although without treatment, the mortality was more than 70% and has now decreased to <20% [32], many of the survivors have persisting neurological deficits. The prognosis of other viral encephalitides is generally comparable to that of HSV encephalitis [32].

### Treatment of viral infections of the nervous system

#### Supportive therapy is the mainstay of treatment of viral nervous system infections

Neurologic and systemic complications may exacerbate brain damage and should be identified and treated early with supportive therapy to optimize neurologic recovery (Table 1). Evidence from RCTs is lacking and thus corticosteroids should not be used routinely; they may be used in selected cases with significant edema, in postinfectious encephalitis and in VZV encephalitis [32].

#### Early administration of antivirals is key for treatment of herpetic viral infections

The drug of choice for the treatment of HSV encephalitis is high-dose intravenous acyclovir which should be administered as early as possible for 14 to 21 days. A clinical trial is currently assessing longer courses of therapy using oral valacyclovir [32]. There are no clinical trials regarding the use of antivirals for VZV encephalitis [32] but acyclovir for up to 3 weeks is recommended for severe infections like encephalitis. A longer course of therapy may be considered for immunocompromised patients. Foscarnet is the preferred agent against HHV-6 whereas combination therapy with foscarnet and ganciclovir is recommended as initial treatment of CMV encephalitis (Table 1).

#### The use of antivirals is limited in non-herpetic viral nervous system infections

Antivirals have not been proven effective for enterovirus encephalitis. The drug pleconaril is an inhibitor of viral replication and may be an option for patients with severe Enterovirus infections [32]. Use of oseltamivir is appropriate for severe influenza. There is also no specific treatment for most causes of encephalitis although experimental therapies may be considered [13, 113].

### Viral causes of shock in the ICU

#### Viral myocarditis can cause cardiogenic shock

Numerous viruses can cause viral myocarditis, including Coxsackie viruses group A and B (Table 1) [41]. Most patients recover, but persistent cardiac dysfunction is associated with 20% one-year mortality [199]. The majority of patients with acute myocarditis have evidence of heart failure. In severe cases mechanical ventricular assist device support is necessary until resolution or cardiac transplantation is available [199]. Although immunosuppressive medicines including corticosteroids were applied in many studies with viral myocarditis, meta-analyses have shown that their effects remain controversial since they do not reduce mortality [37]. In a systematic review, the use of intravenous immunoglobulins (IVIGs) in viral myocarditis was not recommended [38]. Experimental strategies for treatment of viral myocarditis have been developed [13, 113, 200].

#### Viral Hemorrhagic Fevers (VHF) can cause distributive shock

Viral hemorrhagic fevers (VHF) are caused by RNA viruses. The main vectors involved in transmission are and rodents or arthropods (Table 1). The clinical syndrome of hemorrhagic fever is secondary to capillary leakage due to increased vascular permeability. Other clinical manifestations depend on the virus involved and include, hepatitis, encephalitis, and/or nephropathy as well as multiorgan failure. Disseminated intravascular coagulopathy (DIC) is one of the common characteristic findings to many but not all of these viruses. There is a wide range of case-fatality rates that may vary from 1% to 90% [113]. Immediate isolation is critical for effective infection control and prevention of transmission in suspect cases. Close collaboration with local and national public health authorities is necessary to alert the community of a possible outbreak [113]. Since there are no effective therapeutic interventions for most of the viruses the care is largely supportive. No corticosteroids should be used. There are no antiviral drugs available for the treatment of hemorrhagic fever viruses, and there is only one vaccine widely available, i.e. the yellow fever 17D vaccine. Ribavirin has been reported to be an effective therapy for Lassa fever [49], but not against other hemorrhagic fever virus infections in humans [113]. Specific immune human plasma has been successful in treating certain hemorrhagic fevers such as the Argentinian hemorrhagic fever [42]. Emerging therapies with activity against VHF including Ebola have been described and are under development [13, 113, 201, 202].

### Other important considerations regarding treatment of viral infections in the ICU

#### Infection control measures have a major role in the management of viral infections in the ICU

The primary factor responsible for transmission of viral infections in the ICU seems to be inadequate training in or compliance with infection control procedures [203, 204]. The use of nebulizers, open suctioning of respiratory secretions, the use of Bi-PAP, endotracheal intubation, outdated ventilation systems may also lead to spread of viral infections in the ICU setting [203, 204]. Infection control measures should include airborne, droplet and contact precautions. Disinfectants are highly active against many viruses [203, 204].

#### Vaccines are not adequate in preventing the spread of many viral infections in the ICU

Vaccination is possible to prevent infections with some viruses: influenza A and B viruses, HBV, varicella-zoster virus, Yellow fever virus and poliovirus. However vaccines are not available for major viral infections such as herpes simplex virus (HSV) and antiviral therapy is needed to control viral infections that cannot be prevented by vaccination.

#### Numerous antiviral drugs are undergoing clinical trials

The emergence of resistant viruses underlines the need to find novel antiviral. A few novel strategies have been introduced for antiviral research but further research is needed before they can be used for treatment of drug-resistant viral infections [13, 113].

#### Targeting latency may lead to complete treatment of chronic latent infections

Despite effective antiviral therapy for certain chronic viral infection (e.g. Hepatitis B), the virus can integrate its genome into the host cell and become latent. Therefore, new therapies that can completely remove viral components integrated in host cells are needed [13, 113].

## Conclusion

Patients with severe viral infections are often hospitalized in intensive care units (ICUs). Viral infections can cause severe morbidity and mortality in certain hosts (Table 1) [4–6]. The mainstay of therapy of viral infections is supportive care. Antiviral therapy is available for a limited number of infections including influenza and herpetic infections. Novel antiviral treatments that target viral proteins (mostly involved in enzymatic activities or in the viral replication machinery) or host proteins that regulate immunity or other cellular processes in host cells and are involved in the viral life cycle need to be further validated in animal and human randomized controlled studies.
